# Effect of experimental orthodontic pain on gray and white matter functional connectivity

**DOI:** 10.1111/cns.13557

**Published:** 2020-12-28

**Authors:** Feifei Zhang, Fei Li, Hong Yang, Yu Jin, Wenli Lai, Neil Roberts, Zhiyun Jia, Qiyong Gong

**Affiliations:** ^1^ Huaxi MR Research Center (HMRRC) Department of Radiology, Functional and Molecular Imaging Key Laboratory of Sichuan Province, West China Hospital of Sichuan University Chengdu China; ^2^ Research Unit of Psychoradiology Chinese Academy of Medical Sciences Chengdu China; ^3^ State Key Laboratory of Oral Disease Department of Orthodontics West China School of Stomatology, Sichuan University Chengdu; ^4^ School of Clinical Sciences University of Edinburgh Edinburgh UK; ^5^ Department of Nuclear Medicine West China Hospital of Sichuan University Chengdu China

**Keywords:** functional connectivity, gray matter, orthodontic pain, Psychoradiology, resting‐state functional magnetic resonance imaging, white matter

## Abstract

**Aim:**

Over 90% of patients receiving orthodontic treatment experience clinically significant pain. However, little is known about the neural correlates of orthodontic pain and which has therefore been investigated in the present study of healthy subjects using an experimental paradigm.

**Methods:**

Resting‐state functional magnetic resonance imaging (rsfMRI) was performed in 44 healthy subjects 24 hours after an elastic separator had been introduced between the first and the second molar on the right side of the lower jaw and in 49 age‐ and sex‐matched healthy control (HC) subjects. A K‐means clustering algorithm was used to identify functional gray matter (GM) and white matter (WM) resting‐state networks, and differences in functional connectivity (FC) of GM and WM between the group of subjects with experimental orthodontic pain and HC were analyzed.

**Results:**

Twelve GM networks and 14 WM networks with high stability were identified. Compared with HC, subjects with orthodontic pain showed significantly increased FC between WM12, which includes posterior thalamic radiation and posterior cingulum bundle, and most GM networks. Besides, the WM12 network showed significant differences in FC with three GM‐WM loops involving the default mode network, dorsal attention network, and salience network, respectively.

**Conclusions:**

Orthodontic pain is shown to produce an alteration of FC in networks relevant to pain processing, which may be mediated by a WM network relevant to emotion perception and cognitive processing.

## Graphical abstract

The orthodontic pain is an uncomfortable and dull orofacial pain caused by tooth movement during the orthodontic treatment. Nearly more than 90% of orthodontic patients suffer from orthodontic pain. However, little is known about the comprehensive effects of orthodontic pain on brain function in large‐scale networks and the functional role of white matter (WM) in the neural mechanism of pain is still not clear. The present study is to investigate functional connectivity (FC) changes in gray matter (GM) and WM networks and interaction between them in subjects with orthodontic pain compared to healthy controls by using resting‐state functional magnetic resonance imaging (fMRI). A K‐means clustering algorithm was used to identify functional GM and WM networks, and differences in FC of GM and WM between the group of subjects with experimental orthodontic pain and controls were analyzed. In the present study, 44 subjects were recruited to the orthodontic pain group and 49 subjects to the control group. We found there were 3 loops connecting GM and WM with altered FC between groups due to the effects of orthodontic pain: i) default mode network (DMN)‐posterior thalamic radiation‐inferior parietal WM network‐DMN; ii) dorsal attention network (DAN)‐posterior thalamic radiation‐middle frontal network‐DAN; iii) salience network (SN)‐posterior thalamic radiation‐middle frontal and middle cingulum bundle network‐SN. The findings, with which WM networks mediate orthodontic pain related sensations and experience, may guide the development of methods for pain relief in clinical orthodontic pain in the future.

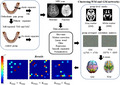

## 1 INTRODUCTION

Orthodontic pain is an uncomfortable and dull orofacial pain produced by orthodontic treatment such as inserting a separator to produce tooth movement.^1^ More than 90% of orthodontic patients suffer from orthodontic pain.^2^, ^3^ It is thought that pain signals arising in the mandible are transmitted to the limbic system, hippocampus and somatosensory cortex via the trigeminal ganglion, trigeminal caudate nucleus and pathways ascending from the thalamus.^4^ In a previous study in this laboratory,^5^ compared to healthy controls, the amplitude of low‐frequency fluctuation (ALFF) was significantly increased in the left insula and right supplementary motor area, and decreased in the right angular gyrus, left precuneus, and superior frontal gyrus in patients with orthodontic pain. Furthermore, the gray matter (GM) functional connectivity (FC) between the left precuneus and left middle temporal gyrus was found to be negatively correlated with scores of pain intensity.

Psychoradiology is an emerging subspecialty of radiology, and it's rapid development has led to our better understanding of the complex brain abnormalities in patients with Pain^6^, ^7^, ^8^.

Numerous studies have shown that pain can produce highly significant changes in brain structure and function. The brain structure that is reported to be most affected is the anterior cingulate cortex (ACC),^9^, ^10^, ^11^, ^12^ and inhibition of the functional activity of ACC can relieve pain^13^, ^14^ and unpleasant pain‐related emotion.^15^ Pain also produces activity in the posterior cingulate cortex (PCC),^16^, ^17^ and FC between PCC and right temporoparietal junction is reported to be highly significant reduced in patients with pain symptoms.^18^ These two cortices are key nodes of the default mode network (DMN) and the salience network (SN), respectively. In patients with trigeminal neuralgia, the functional activity of DMN is decreased,^19^ and FC between PCC and middle prefrontal cortex is weakened.^20^ Moreover, pain has also been shown to produce alterations in white matter (WM). There is compelling evidence that fractional anisotropy measured by diffusion‐weighted imaging is significantly decreased in the corpus callosum and corona radiata in patients with trigeminal neuralgia.^21^ Furthermore, the altered microstructure of the corpus callosum^22^ and corona radiata^22^, ^23^ was significantly correlated with pain intensity. Other WM regions, such as the parahippocampal sections of the cingulum bundle,^24^, ^25^ tapetum,^24^ internal and external capsule,^22^, ^26^ and thalamic radiation,^22^, ^27^ have been reported in patients with pain symptoms. However, there has been no study of the functional role of WM in the neural mechanism of pain.

Originally, the blood oxygenation level‐dependent (BOLD) signal of resting‐state functional magnetic resonance imaging (rsfMRI) data obtained for WM was considered to be noise. However, new techniques have demonstrated that the BOLD signals within WM can reflect intrinsic neural activity and carry important information.^28^, ^29^, ^30^, ^31^, ^32^ The objective of the present study was to use rsfMRI investigating whether there were changes in the FC of GM and WM networks and the interaction between them in subjects with experimentally induced orthodontic pain compared to matched controls. The hypothesis that FC between pain‐related cortex (eg, DMN and SN) and WM (eg, thalamocortical radiation) would show significant alterations in the early phase of orthodontic pain was tested. This study will provide a comprehensive understanding of the neural mechanism of orthodontic pain from the perspective of FC between GM and WM.

## 2 MATERIALS AND METHODS

### 2.1 Participants

This study was approved by the Research Ethics Committee of West China Hospital, Sichuan University, and fully informed written consent of their willingness to participate was obtained from all subjects. All 48 subjects were recruited to the orthodontic pain group and 49 subjects to the control group. All subjects were aged between 18 and 30 years, right‐handed, of Chinese Han nationality, and had no history of receiving orthodontic treatment. Exclusion criteria were (i) any history of major illness, such as central nervous system disorders, cardiovascular diseases, and severe head trauma with loss of consciousness, (ii) other diseases causing orofacial pain, including gingivitis and periodontitis, (iii) have malocclusion, (iv) substance or non‐substance addiction, and (v) claustrophobia, pregnancy, and any contraindications for MRI to be performed.

### 2.2 Experimental protocol

According to previous findings, orthodontic pain generally occurs within 12 hours of treatment, reaches a peak after about 24 hours, and then gradually subsides over 3 to 7 days, finally returning to baseline over a month.^3^, ^33^ For each subject in the orthodontic pain group, the elastic orthodontic separator was inserted between the first and the second molar on the right side of the lower mandible for 24‐hours by an experienced dentist. The elastic orthodontic separator was inserted with the aid of two dental floss segments. We stretched the separator with two pieces of dental floss and pulled it toward the gingiva between the two teeth. Half of the separator was below the contact area, and the other half was above the contact area.^34^ Twenty‐four hours later, MRI scanning was performed with the elastic separator still in‐site, which was removed immediately after the scanning.

The subjects completed the State Anxiety Scale (SAI) questionnaire^35^ and visual analogue scale (VAS) recording their perception of anxiety and pain intensity. All measurements were tested twice, the first before insertion of the elastic separator, and the second 24 hours after insertion of the elastic separator and before MRI scanning. The elastic separator was not inserted, and measurements of SAI and VAS were not obtained for the control group.

### 2.3 Data acquisition

MR investigations were performed using a Siemens 3T system (Tim Trio, Siemens Healthineers, Erlangen, Germany) equipped with an eight‐channel head coil. The rsfMRI data were collected using an echo‐planar imaging (EPI) sequence with the following acquisition parameters of repetition time (TR) 2 seconds, echo time (TE) 30 ms, flip angle 90°, slice thickness 5 mm, matrix 64 × 64, a field of view (FOV) 240 mm × 240 mm corresponding to a voxel size of 3.75 mm × 3.75 mm × 5 mm. A total of 205 volumes each comprising 30 contiguous slices were acquired in a total imaging time of 6 minutes 50 seconds. During MRI scanning, participants were asked to close their eyes and try to avoid thinking about a particular subject. A 3D T1‐weighted image was also obtained for each subject using a magnetization‐prepared rapid gradient‐echo (MPGR) sequence with acquisition parameters of TR 1.96 seconds, TE 2.26 ms, flip angle 90°, matrix 256 × 256, and FOV 240 mm × 240 mm for 175 contiguous 1‐mm‐thick sections. The images were reviewed by a radiologist to assess whether there were any signs of gross brain lesions on the T1‐weighted images.

### 2.4 Imaging processing

The MRI data were processed using SPM8 (www.fil.ion.ucl.ac.uk/spm), DPARSFA (rfmri.org/DPARSF), and MATLAB (www.mathworks.com) software. The 3D T1‐weighted images were segmented into compartments of GM, WM, and cerebrospinal fluid (CSF) using the new segment algorithm in SPM8,^36^ and the rsfMRI data were processed in DPARSFA using the following processes: (i) The first 10 volumes were removed to ensure equilibrium of the magnetization, and a slice timing correction was performed, (ii) using the middle image as a reference slice, the images were corrected for head motion and subjects with translation >2.0 mm or rotation >2.0° were excluded, (iii) the corrected rsfMRI images were co‐registered with the 3D T1‐weighted anatomical image, (iv) followed by linear detrending and filtering (0.01‐0.1 Hz), (v) regression out of nuisance signal via a 24‐parameter motion correction and analysis of the mean signal of CSF,^37^ (vi) removal of motion “spikes” with a high framewise displacement >1 mm^38^ based on a separate regression which did not include analysis of the signals from WM and whole brain,^31^, ^39^ (vii) separate smoothing of signals from GM and WM using a filter with full width half maximum of 4 mm,^31^ and (viii) normalization of the processed functional images resampled to a voxel size of 3 mm × 3 mm × 3 mm to the standard Montreal Neurological Institute (MNI) EPI template.

### 2.5 Clustering WM and GM networks

To further analyze the rsfMRI data, the first step was to obtain the group‐level GM and WM masks from the high‐resolution T1‐weighted images. For each subject, the T1‐weighted 3D images have been segmented to GM, WM, and CFS according to the maximum probability method. Then, these masks were averaged among subjects to obtain the percentage of each voxel that was classified as WM or GM. A threshold value of 60% was used to define the WM mask, and a value of 20% was used to define the GM mask with any voxels contained in the WM mask excluded.^31^ Using the Harvard–Oxford Atlas,^40^ deep gray nuclei which had been incorrectly incorporated were removed from the WM mask and labeled as GM. This included the thalamus, caudate nucleus, putamen, globus pallidus, and nucleus accumbens. The resulting group‐level GM mask comprised 42,795 voxels and a group‐level WM mask comprised 19,576 voxels were co‐registered to the MNI‐EPI template, and the following analyses referred to the restricted regions corresponding to these masks. There was no overlap between the GM and WM masks.

To create group‐averaged WM correlation matrices for clustering the WM networks, we calculated the Pearson correlation coefficient between each WM voxel and the other WM voxels. Firstly, we used the inter‐changing grid to resample the WM mask, extracted any second voxels along the rows and columns of the image, and moved 1 between slices to avoid losing the entire data column. Then, for each WM voxel, the correlation with all sub‐sampled mask nodes was calculated to obtain the whole‐brain correlation pattern of each voxel (19 576 × 4895 matrices).^41^ The average matrix was taken from all 93 subjects’ correlation matrices to obtain a group‐level correlation matrix. To exclude GM signal, for the correlation between voxels, the data used to average across subjects were only taken from subjects whose two voxels were identified as WM during segmentation. Vice versa for the correlation matrices of GM (42,795 × 4759 matrices).

To identify the GM and WM functional networks, K‐means clustering (distance metric‐correlation, 10 replicates)^31^ was performed on the rows of the average group‐level correlation matrix for the GM and WM, respectively. In this way, the GM or WM voxel clusters with similar connection patterns to other voxels can be identified. All clusters of sizes between 2 and 22 were analyzed with coarse and fine granularity to determine the most stable value for the number of networks.^31^

To identified the most stable of the K (the number of clusters), we measured the stability of the clustering solution for each number of clusters, from 2 to 22.^41^ The full connectivity matrix was randomly divided into 4 folds, with each fold containing the correlations between all voxels at the quarter sub‐sampled masks (19,576 × 1224 voxels per fold for WM; 42,795 × 1190 voxels per fold for GM). A separate clustering of between 2 and 22 was performed for each of the 4 folds. According to different characteristics, the clustering results should be approximately similar for the number of clusters representing stable clustering solutions.^41^ Therefore, the adjacency matrix for each solution was calculated to measure the similarity between clustering solutions, and the Dice coefficient was used to compare these adjacency matrices. For each cluster number, the average Dice coefficient was obtained from all four adjacent matrices. A K value with a Dice coefficient of greater than 0.85 was considered a stable solution.^38^

### 2.6 Statistical analysis

Pearson's correlation coefficient was computed between the average time courses of pairs of networks and used to construct K_Gray_ × K_Gray,_ K_White_ × K_White,_ and K_Gray_ × K_White_ matrix for each subject.^32^ The FC value was transformed into the Fisher *z* score. Then, we calculate the kurtosis and skewness of all matrices, and the results show that all the FC values are between −2 and 2, indicating that the data were confirmed to be normally distributed.^42^ A one‐sample t‐test was then performed to determine the significant FC for each group. Subsequently, for these significant FC, two‐sample *t*‐tests were performed to compare the differences between the orthodontic pain group and controls. Finally, partial correlation analysis was performed to examine the relationship between network FC and differences in SAI and VAS scores measured at baseline and 24 hours after the application of the elastic separator in the orthodontic pain group, with age and sex used as covariates. Bonferroni correction for multiple comparisons was used for the FC and correlation analyses with statistical significance level at *P* < 0.05.

## 3 RESULTS

### 3.1 Demographic and clinical characteristics

In subjects with pain, three subjects were excluded on account of head motion exceeding 2.0 mm in translation or 2.0° in rotation during fMRI scanning and one was excluded because of a potential mental illness. So that the orthodontic pain group comprised 44 subjects (24 females and 20 males, mean age 21.0 ± 0.9 years, range 19 to 23 years) and the control group comprised 49 subjects (27 females and 22 males, mean age 21.0 ± 2.6 years, range 19 to 30 years) for the final analyses. There were no significant differences in age (*P* = 0.958) or sex (*P* = 0.991) between the two groups. Paired t‐tests revealed that there was no significant change in SAI (*P* = 0.21) but a significant increase in VAS pain intensity score (*P* = 0.01) 24 hours after the insertion of the elastic separator in subjects in the orthodontic pain group. (Table 1).

**TABLE 1:** Demographic and clinical characteristics of participants with orthodontic pain (ORTH) and healthy controls (HCs)

Characteristics	ORTH (n = 44)					HCs (n = 49)	*P* values
*Demographic factors*							
Sex (male/female)	20/24					22/27	0.95
Age (years)		21.0 ± 0.9				21.0 ± 2.6	0.99
		Without separator		24 h after with separator			
Visual analogue scale		13.66 ± 16.35		20.48 ± 18.09		‐	0.01
		**Skewness**	**Kurtosis**	**Skewness**	**Kurtosis**		
		**‐0.92**	**0.43**	**‐0.88**	**‐0.28**		
State Anxiety Scale		27.73 ± 11.00		29.82 ± 10.48		‐	0.21
		**Skewness**	**Kurtosis**	**Skewness**	**Kurtosis**		
		**0.02**	**‐0.61**	**0.17**	**‐0.53**		

### 3.2 GM and WM networks

**Figure 1:**
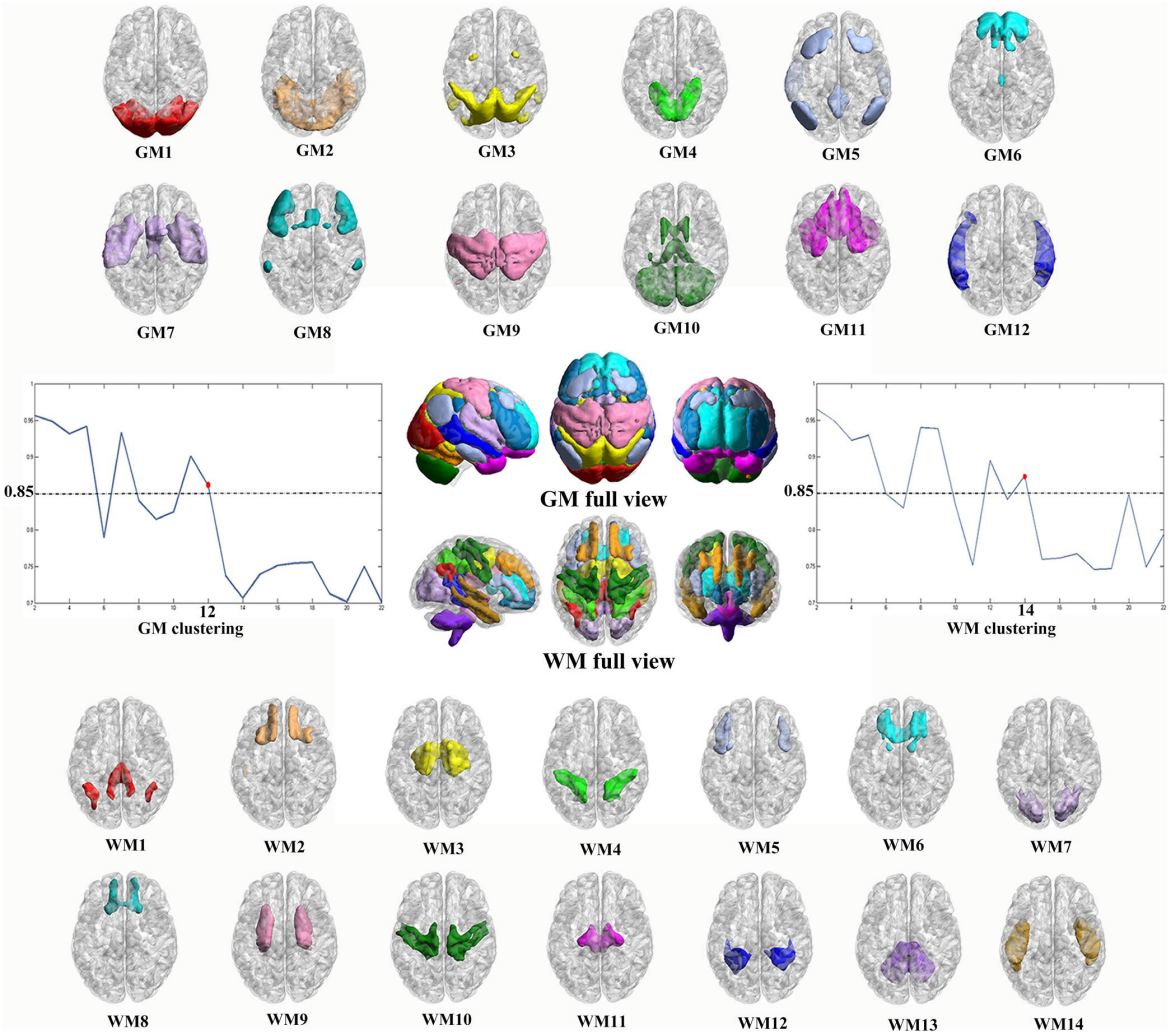
Twelve gray matter (GM) networks and 14 white matter (WM) networks were found to have a high stability as measured by Dice coefficient in K‐means clustering analysis

The K‐means clustering analysis revealed there to be 12 GM networks with high stability were defined as lateral visual network (GM1), anterior lobe of cerebellum network (GM2), dorsal attention network (DAN) (GM3), medial occipital network (GM4), DMN (GM5), superior frontal network (GM6), SN (GM7), executive control network (ECN) (GM8), somatomotor network (GM9), posterior lobe of the cerebellum and subcortical network (GM10), orbitofrontal–temporal network (GM11), and middle temporal network (GM12) (Figure 1).

The same K‐means approach revealed there to be 14 WM networks with high stability which were named as posterior cingulum (retrosplenial) bundle and angular WM network (WM1), inferior frontal WM network (WM2), corona radiata network (WM3), inferior parietal WM network (WM4), middle frontal WM network (WM5), anterior cingulum bundle network (WM6), occipital WM network (WM7), orbitofrontal WM network (WM8), middle cingulum bundle network (WM9), precentral/postcentral WM network (WM10), brainstem network (WM11), posterior thalamic radiation and posterior cingulum bundle network (WM12), cerebellum WM network (WM13), and inferior longitudinal fasciculus network (WM14) (Figure 1), which was consistent with previous studies.^31^, ^38^

### 3.3 Alterations in FC between GM networks

**Figure 2:**
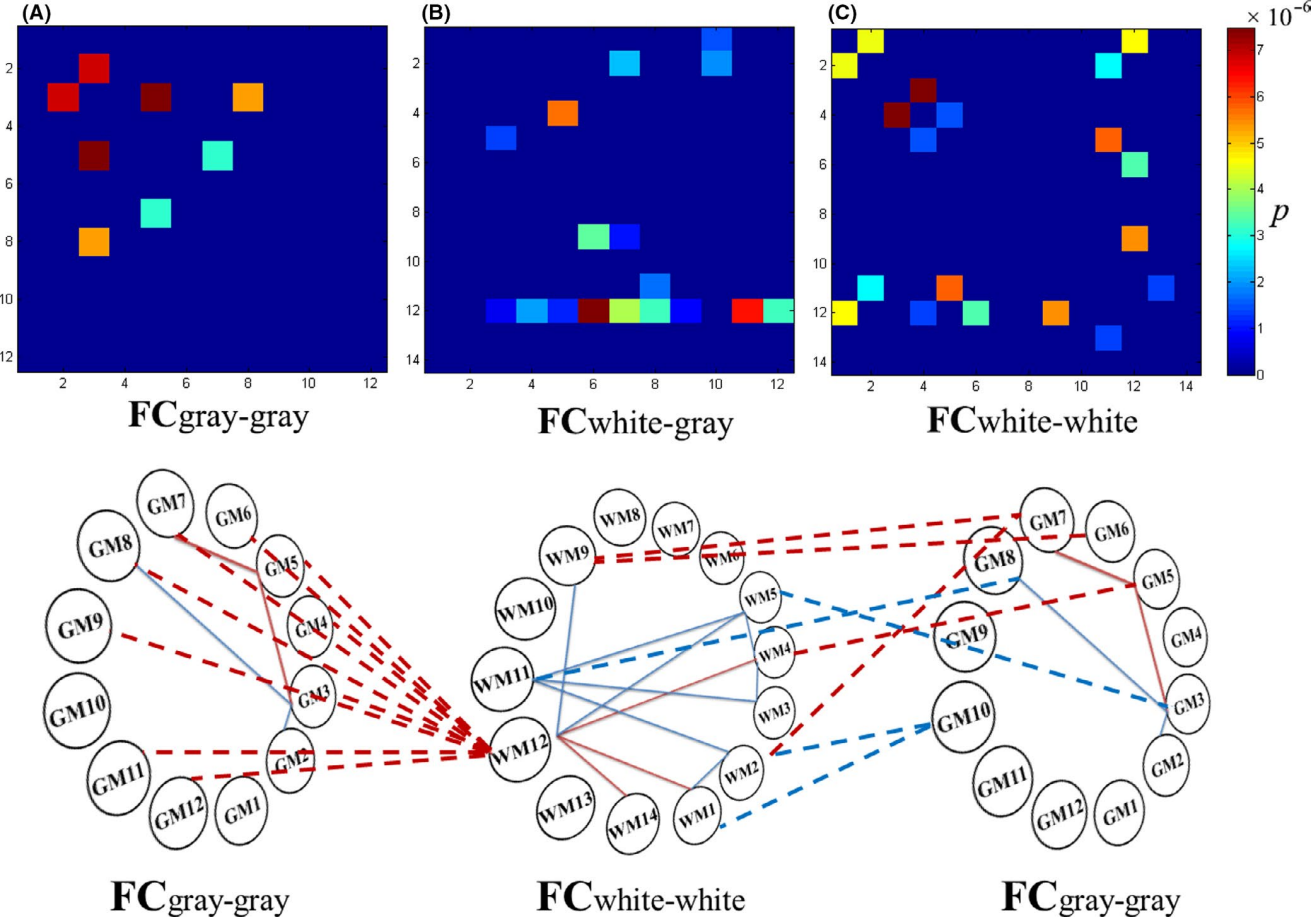
Three matrices showed significantly altered functional connectivity (FC) in the orthodontic pain group compared with controls: (A) FC between gray matters (GM) networks, (B) FC between GM and white matter (WM) networks, and (C) FC between WM networks. The values in the graph were represented by *P* values. The solid lines indicate altered FC between 12 GM networks and between 14 WM networks, respectively, whereas the dotted lines indicate altered FC between the GM and WM networks in orthodontic pain group. Red and blue lines, respectively, represent increased and decreased FC in subjects with orthodontic pain compared with controls,

Compared with the control group, participants in the orthodontic pain group showed higher FC between GM3 and GM5 and between GM5 and GM7 (for a concise description, we abbreviated them as GM3‐GM5‐GM7, which corresponds to the well known DAN‐DMN‐SN networks, and the following texts were similar), and lower FC in GM2‐GM3‐GM8 (Figure 2A).

### 3.4 Alterations in FC between WM networks

Compared with the control group, participants in the orthodontic pain group showed increased FC between WM12 and three other WM networks, including WM1, WM4, and WM14, and decreased FC in two pathways, namely, WM1‐WM2‐WM11‐WM3‐WM4‐WM5‐WM11 and WM5‐WM12‐WM9 (Figure 2C).

### 3.5 Alterations in FC between GM and WM networks

The effects of orthodontic pain on the brain were also reflected in FC between GM and WM networks. In particular, compared to the control group, the orthodontic pain group showed increased FC between GM5 and WM4 and in GM6‐WM9‐GM7‐WM2. Furthermore, FC was increased between WM12 (posterior thalamic radiation and posterior cingulum bundle network) and 9 GM networks (ie, all GM networks except for GM1, GM2, and GM10 representing lateral occipital areas, cerebellum, and subcortical areas, respectively). Besides, compared to the control group, the orthodontic pain group showed decreased FC between GM3 and WM5, between GM8 and WM11, and in WM1‐GM10‐WM2 (Figure 2B).

### 3.6 Three GM and WM networks loops with altered FC

**Figure 3:**
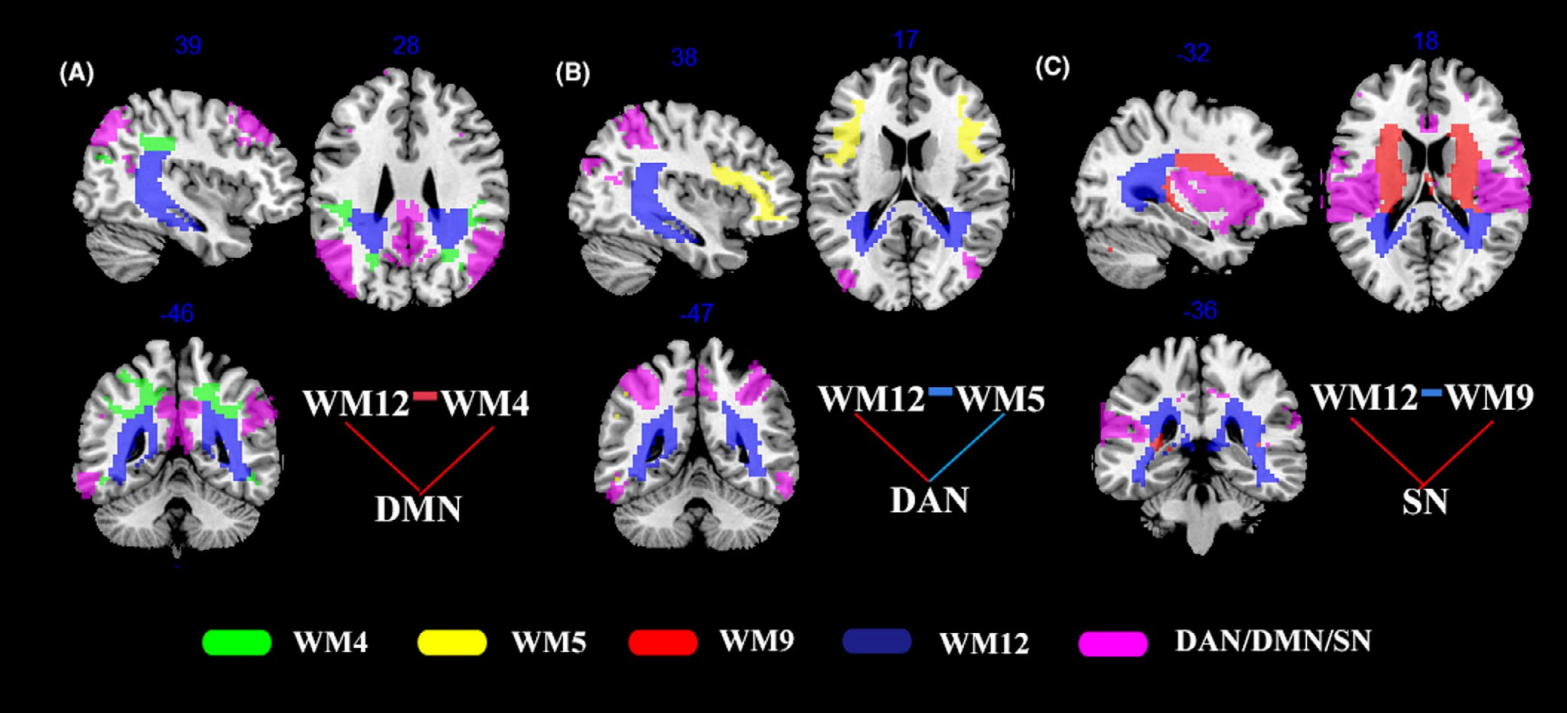
Three circuits between gray matter (GM) networks (shown in purple) and white matter (WM) networks that are altered in the orthodontic pain group compared to controls, including (A) the default mode network (DMN)‐WM12‐WM4‐DMN, (B) dorsal attention network (DAN)‐WM12‐WM5‐DAN, and (C) salience network (SN)‐WM12‐WM9‐SN. Red and blue lines drawn between different networks, respectively, represent increased and decreased FC in subjects with orthodontic pain compared to controls

Further consideration of the above findings, there were significant alterations in FC between GM and WM networks in the orthodontic pain group compared to controls corrected for multiple comparisons with Bonferroni method and summarized to three loops (ie, combination of three or more GM and WM networks with at least one network in GM and one network in WM), namely, (i) a loop corresponding to GM5‐WM12‐WM4‐GM5 (ie, DMN‐posterior thalamic radiation‐inferior parietal WM network‐DMN) with increased FC in all connections (Figure 3A), (ii) a loop corresponding to GM3‐WM12‐WM5‐GM3 (ie, DAN‐posterior thalamic radiation‐middle frontal network‐DAN), in which FC between DAN and WM12 was increased, while FC was decreased in the two other connections (Figure 3B), and (iii) a loop corresponding to GM7‐WM12‐WM9‐GM7 loop, where there was increased FC between WM and GM networks and decreased FC between the posterior thalamic radiation and posterior cingulum bundle network (WM12) and the middle cingulum bundle network (WM9) (Figure 3C).

### 3.7 Correlation between pain score and FC networks

Analysis of the relationship between significantly increased pain scores and FC in the orthodontic pain group revealed a negative relationship between VAS score recorded immediately before the MRI scanning (ie, 24 hours after insertion of the elastic actuator) and FC between GM6 and WM9 (*r* = −0.309, *p*_uncorrected_ = 0.047) and GM4‐WM12 (*r* = −0.311, *p*_uncorrected_ = 0.045). However, neither result survived the correction for multiple comparisons.

## 4 DISCUSSION

Analysis of rsfMRI data has shown significant alterations in brain FC of both WM and GM networks in subjects with experimentally induced orthodontic pain compared with control subjects. Fourteen WM and 12 GM networks were shown to have high stability by K‐means analysis, and there were two main findings regarding the FC of these networks. Firstly, a WM network comprising posterior thalamic radiation and posterior cingulum network (WM12) showed increased connections with almost all GM networks in the orthodontic pain group. Secondly, the FC of three loops connecting GM and WM was significantly altered in the group of subjects with orthodontic pain, namely, (1) DMN‐WM12‐WM4‐DMN, (2) DAN‐WM12‐WM5‐DAN, and (3) SN‐WM12‐WM9‐SN loops. Until recently, there have been only a few studies of WM functional networks, the existence of which was not well accepted or considered to be of interest. Alterations in WM network FC are a prominent aspect of both the main findings of the present study.

### 4.1 Increased FC between posterior thalamic radiation and GM networks

Alterations in the FC of the WM network comprising posterior thalamic radiation and posterior cingulum network (ie, WM12) were prominent in both the main findings of this study of experimental orthodontic pain. The thalamus is a main component of the so‐called pain matrix and is activated by the perception of orthodontic pain and its modulation.^1^, ^43^ This central role arises from the fact that there are structural connections between the thalamus and the GM of all regions of the neocortex,^44^ and accordingly, the thalamus is involved in integrating and modulating signals from widespread brain regions and especially those involved in processing the sensory and emotional qualities of pain.^45^ The observation of increased FC between WM12 and nine GM networks reveals a potential mechanism whereby pain signals produced in the thalamus can be transmitted via the thalamic radiation to other brain regions involved in pain processing^1^ such as the amygdala, insula, hippocampus, and sensory‐motor cortex and so amplify feelings of pain produced by the orthodontic elastic separator.^45^, ^46^

Consistent with a previous pain treatment study revealing that the pain can be successfully suppressed by stimulating the thalamic nucleus,^46^ the negative correlation between pain scores and increased FC of GM4‐WM12 indicated that restoration of the connectivity between the medial occipital GM network (GM4) and posterior thalamic radiation WM network (WM12) may produce pain relief, which could be naturally as a functional compensation or neural adaptation to orthodontic pain.^5^ Therefore, our findings indicated that the part of thalamocortical pathways with increased FC are likely to be involved in the perception of the sensation of pain relief,^45^ and further confirmed the key role of the thalamic radiation in pain transmission and regulation^4^ from the perspective of neuroimaging research.

### 4.2 Altered FC of three loops connecting GM and WM networks

Concerning the second main finding, the FC of three GM‐WM loops was altered as a result of orthodontic pain. The first GM‐WM loop to be discussed comprises DMN‐WM12‐WM4‐DMN and thus involves posterior thalamic radiation (WM12), inferior parietal WM (WM4), and the DMN, between which there was increased FC in subjects with orthodontic pain. Brain activity in DMN has been widely reported to be modulated by changes in pain perception^47^, ^48^ and self‐oriented attention.^48^ Normally, DMN is active during the resting state and becomes deactivated during the performance of a task. Accordingly, it might have been expected that FC of DMN would be decreased in perceiving a painful stimulation.^49^ However, in previous rsfMRI studies, it has been reported that typical pain‐related deactivation of DMN disappeared in patients with pain for whom increased FC of DMN was observed during resting state,^50^, ^51^ consistent with the results of the present study. Since the thalamus is involved in attention and processing of the emotional dimension of pain,^52^ and a positive correlation has previously been reported between rumination and FC in the thalamo‐DMN pathway,^50^ the increased posterior thalamic radiation‐DMN FC in the present study may reflect persistent attempts by subjects in the orthodontic pain group to regulate and manage their feelings of pain. The increased FC between the inferior parietal WM network (WM4) and DMN may reflect excessive attention to orthodontic pain^48^ and a high degree of rumination about the negative effects of pain^50^ in the experimental group.

The second GM‐WM loop to be discussed is DAN‐WM12‐WM5‐DAN. This is interpreted as comprising the main nodes of the dorsal attention network (DAN) in the frontal and parietal lobe, the middle frontal WM network (WM5), and the posterior cingulum bundle which is another important part of WM12, forming a fronto‐cingulo‐parietal circuit with decreased FC within WM12‐WM5‐DAN and increased FC in DAN‐WM12 in subjects of the orthodontic pain group than controls. This interpretation is consistent with the results of a previous pain‐related attention study, in which it was reported that the fronto‐cingulo‐parietal network was functionally disrupted under the influence of pain.^53^ The DAN is likely to be activated in attending to pain sensations, which may be reduced by distraction^54^ with the possibility that the decreased FC within WM12‐WM5‐DAN may be related to pain relief occurring as a result of functional adaptation of the brain to the pain. The additional finding of increased FC between the posterior cingulum bundle of WM12 and DAN in this loop may be explained by a report that the connection between PCC and DAN plays a crucial role in balancing internally and externally directed attention.^55^ In another study, it was reported that the activity of the attention network was the greatest during attention to pain.^48^ Furthermore, Yoshino et al have reported that in addition to FC of cingulo‐DAN being increased as a result of painful stimulation, a negative correlation was subsequently found between cingulo‐DAN FC and decrease in pain intensity after treatment.^56^ The increased FC between WM12 and DAN observed in the present study may indicate that orthodontic pain is characterized by attention to the pain sensation caused by an external stimulus, namely, the elastic separator.

The third GM‐WM loop is GM7‐WM12‐WM9‐GM7. GM7 involves bilateral insula, temporoparietal junction, and ACC, which are key nodes of SN. The middle cingulum bundle (WM9) is also connected with SN and WM12 structurally. In previous studies, increased FC of SN^57^ between insular and PCC^18^ and between insular and thalamus^58^ has been reported in patients with chronic pain. The increased FC of insular–PCC/thalamus was positively correlated with pain duration^58^ and influenced by imbalances in the level of excitatory and inhibitory neurotransmitters.^59^ The excitatory amino acid, glutamate, was found to be increased as a result of painful stimulation,^60^ and it is thus possible that the increase of FC, between insula and PCC and between insula and thalamus, may be caused by hyperactivity of glutamatergic neurons produced as a result of painful stimulation. The present study has added to the above knowledge of increased FC among GM networks, suggesting the WM networks (posterior thalamic radiation and middle cingulum bundle) also having an increased FC with SN under a painful stimulation. Treatment of the emotional, sensory, and perceptual dimensions of pain has been reported to produce reductions in the activity of the posterior and middle cingulum bundle,^55^, ^61^ and pain discomfort has been reported to be successfully reduced by cingulate gyrotomy.^62^ Thus, the decreased FC between WM12 and WM9 and between WM6 and WM9 may represent an adaption to weaken the feelings of pain. However, further studies are required to determine how the alteration in GM‐WM FC contributes to the neurobiological mechanisms in subjects with orthodontic pain.

### 4.3 Limitations

The potential limitations of the study should be considered. Firstly, the rsfMRI analysis only considered the differences in the magnitude of FC between groups and not the direction of the FC within and between GM and WM networks. Future research to provide a more detailed and directional FC analysis will help to elucidate the neurobiological underpinnings of pain arising from orthodontic treatments. Secondly, the experimental paradigm employed in the present study did not simulate the whole process of orthodontic treatment. To further and fully understand the neurological mechanisms that produce orthodontic pain, a longitudinal study is needed and will be made more straightforward if orthodontic treatment is performed using materials that are compatible with and do not produce artifacts on MR images.^63^

## 5 CONCLUSION

The present analysis of rsfMRI data revealed alterations in whole‐brain FC in subjects with experimentally induced orthodontic pain compared to controls, which included increased FC between posterior thalamic radiation and posterior cingulum network (ie, WM12) and most GM networks together with altered FC in three loops of GM‐WM networks that all included WM12. The familiar GM networks of DMN, DAN, and SN were each a prominent feature of respective GM‐WM loops which is interpreted as indicating that there may be an adaptive alteration of FC between GM and WM networks to manage and remediate the debilitating sensations of orthodontic pain. The neuroimaging findings that WM networks mediate orthodontic pain sensations and experience facilitates the understanding of the neural mechanism of orthodontic pain, potentially support the development of effective and comprehensive palliatives and remedies, and may guide the development of methods for pain relief in clinical orthodontic pain in the future.

## CONFLICT OF INTEREST

The authors have no conflict of interest to declare.

## CODE AVAILABILITY

All the codes for analyzing data in this study can be obtained from the corresponding authors upon reasonable request.

## Data Availability

Data supporting the results of this study can be obtained from the corresponding authors upon reasonable request.
